# The Vector Matching Method in Geomagnetic Aiding Navigation

**DOI:** 10.3390/s16071120

**Published:** 2016-07-20

**Authors:** Zhongguo Song, Jinsheng Zhang, Wenqi Zhu, Xiaoli Xi

**Affiliations:** 1 Faculty of Automation and Information Engineering, Xi’an University of Technology, NO.5 South Jinhua Road, Xi’an 710048, China; songzhongguo@xaut.edu.cn (Z.S.); wenqi0508@163.com (W.Z.); 2 Xi’an Research Institute of High Technology, NO.2 Tongxin Road, Xi’an 710025, China; zjinshengchina@163.com

**Keywords:** geomagnetic matching, inertial navigation system, iterative closest contour point, Bayesian theory

## Abstract

In this paper, a geomagnetic matching navigation method that utilizes the geomagnetic vector is developed, which can greatly improve the matching probability and positioning precision, even when the geomagnetic entropy information in the matching region is small or the geomagnetic contour line’s variety is obscure. The vector iterative closest contour point (VICCP) algorithm that is proposed here has better adaptability with the positioning error characteristics of the inertial navigation system (INS), where the rigid transformation in ordinary ICCP is replaced with affine transformation. In a subsequent step, a geomagnetic vector information fusion algorithm based on Bayesian statistical analysis is introduced into VICCP to improve matching performance further. Simulations based on the actual geomagnetic reference map have been performed for the validation of the proposed algorithm.

## 1. Introduction

Geomagnetic matching is a key aiding navigation technology that can rectify the indication trace of the Inertial Navigation System (INS) by comparing the geomagnetic profile acquired on board with the stored geomagnetic map, which is an ideal autonomous navigation candidate for long range Unmanned Aerial Vehicle (UAV) application due to its merits such as the all-weather, whole-day working consistentency [1,2].

There is a kind of scalar matching method often used [3,4,5,6,7,8,9,10,11], which minimizes the square of the differences between the norms of magnetometer outputs and the magnitude of stored geomagnetic reference field in correcting the indication trace. Those methods convert the matching problem into a state estimation problem by using datasets collected in the candidate matching region, most of which are not feasible for long time and long range application because the selection of matching region along the expected trace with high adaptability is difficult, and the estimated algorithm might diverge because of insignificant geomagnetic characteristics [3]. At present, there are three types of scalar geomagnetic matching algorithms: the Extended Kalman Filter (EKF) based algorithm [4,5,6], the Correlation Matching (CM) algorithm [7,8,9] and intelligent algorithm [10]. The conventional EKF and CM based algorithm usually lead to the local optimum solutions, depending on the geomagnetic entropy in the matching region and the matching probability and positioning precision will reduce a lot. The intelligent algorithm generally needs a large number of sample data sets for training, which will result in an inconsistent solution with the small matching region. Recent work in [11] developed a particle filter algorithm based the measurement of static H-field that can only be applied to ground vehicles due to the long time preparation of local magnetic field maps. Due to the development of a low cost, light weight three-axis magnetoresistive magnetometer and flux-gate magnetometer, the onboard measurement accuracy of the geomagnetic vector field has been improved to the order of nanotesla in recent years [12,13]. Therefore, a vector geomagnetic matching method that utilizes the multidimensional geomagnetic element is developed, which aims at obtaining relatively stable and precise positioning performance with geomagnetic terrain uncertainty.

This paper presents a significant improvement upon previous methods which is based on the geomagnetic field vector measurement. The affine transformation, which is often used in machine vision, is adopted to ICCP for the first time to deal with the scale error of INS. The error models of geomagnetic field vector measurement and INS combined effect of all linear time-invariant distortions will firstly be described. Then, the vector matching algorithm, namely Vector Iterative Closest Contour Point (VICCP), is adopted to estimate the trace with an accuracy and robustness solution. Finally, we will present the simulation results for the validation of the proposed algorithms.

## 2. The Error Model of Geomagnetic Vector Matching System

### 2.1. Geomagnetic Field Vector Measurement Error

The error model of geomagnetic field vector measurement combines the effect of all linear time-invariant distortions, which can be described as follows [14]:

(1) $$h_{M}=C_{s}^{e}\left(C_{O}C_{S}\left(D_{SI}h_{E}+D_{HI}\right)+b_{S}\right)+n_{S}$$

where $h_{E}(3\times 1)$ is the error-free geomagnetic field in the sensor frame, and whereas $h_{M}(3\times 1)$ is the readings from the triad of magnetometer in the earth frame. The triangular matrix $C_{O}(3\times 3)$ stands for the nonorthogonality of three-axis magnetometer. The diagonal matrix $C_{S}(3\times 3)$ is scale factor and $b_{s}(3\times 1)$ is the zero offset of the magnetometer. $D_{HI}(3\times 1)$ and $D_{SI}(3\times 3)$ stand for the hard magnetic iron and soft magnetic iron interference respectively. $C_{s}^{e}(3\times 3)$ is external attitude information of the vehicle. $n_{S}$ is Gaussian noise following ~*N*(0, $\sigma_{n}^{2}$). After introducing matrix $C_{M}(3\times 3)$ and vector $d_{M}(3\times 1)$, the error model can be simplified as:

(2) $$h_{M}=C_{M}h_{E}+d_{M}+n_{S}$$

where

(3) $$\{\begin{array}{l} C_{M}=C_{s}^{e}C_{O}C_{S}D_{SI}\\ d_{M}=C_{s}^{e}\left(C_{O}C_{S}D_{HI}+b_{S}\right) \end{array}$$

$C_{M}$ and $d_{M}$ are a linear combination of above-mentioned errors, which can be determined prior to its application or by onboard calibration.

### 2.2. Error Model of INS

Since an INS is available in this paper, we will use an INS error propagation model given as follows [15,16].

(4) $$\dot{x}(t)=F_{INS}x(t)+w(t)$$

omitting the time variable *t*, then $x={\left[\begin{array}{ccccccc} {\left(\phi \right)}^{T} & {\left(\delta v^{n}\right)}^{T} & {\left(\delta p\right)}^{T} & {\left(\varepsilon^{b}\right)}^{T} & {\left(\nabla^{b}\right)}^{T} & {\left(\delta K_{g}\right)}^{T} & {\left(\delta K_{a}\right)}^{T} \end{array}\right]}^{T},$ where platform misalignment angles $\phi ={\left[\begin{array}{ccc} \phi_{E} & \phi_{N} & \phi_{U} \end{array}\right]}^{T}$, velocity errors $\delta v^{n}={\left[\begin{array}{ccc} \delta v_{E}^{n} & \delta v_{N}^{n} & \delta v_{U}^{n} \end{array}\right]}^{T}$ and $\delta p={\left[\begin{array}{ccc} \delta L & \delta \lambda & \delta h \end{array}\right]}^{T}$ with δL,δλ,δh represent latitude, longitude and altitude errors respectively. $\varepsilon^{b}={\left[\begin{array}{ccc} \varepsilon_{x}^{b} & \varepsilon_{y}^{b} & \varepsilon_{z}^{b} \end{array}\right]}^{T}$ is gyro drift errors expressed in body frame and $\nabla^{b}={\left[\begin{array}{ccc} \nabla_{x}^{b} & \nabla_{y}^{b} & \nabla_{z}^{b} \end{array}\right]}^{T}$ is accelerometer biases expressed in body frame. $\delta K_{g}$ can be represented as: $\delta K_{g}={\left[\begin{array}{ccccccccc} \delta k_{gxx} & \delta k_{gyx} & \delta k_{gzx} & \delta k_{gxy} & \delta k_{gyy} & \delta k_{gzy} & \delta k_{gxz} & \delta k_{gyz} & \delta k_{gzz} \end{array}\right]}^{T}$, where $\delta k_{gii},(i=x,y,z)$ are gyro scale factor errors and $\delta k_{gij},(i,j=x,y,z,i\neq j)$ are gyro actual axis misalignment angles with respect to ideal body frame axis. $\delta K_{a}$ can be represented as: $\delta K_{a}={\left[\begin{array}{cccccc} \delta k_{axx} & \delta k_{ayx} & \delta k_{azx} & \delta k_{ayy} & \delta k_{azy} & \delta k_{azz} \end{array}\right]}^{T}$, $\delta k_{aii},(i=x,y,z)$ are accelerometer scale factor errors and $\delta k_{aij},(i,j=x,y,z,i\neq j)$ are accelerometer actual axis misalignment angles with respect to ideal body frame axis. The components $\varepsilon^{b}$, $\nabla^{b}$, $\delta K_{g}$, $\delta K_{a}$ are all assumed to be constant vectors. w(t) are white Gaussian noise sources including the first-order Gauss-Markov bias process for the accelerometer and gyro.

The $F_{INS}$ in Equation (4) is expressed as:

(5) $$F_{INS}=\left[\begin{array}{ccccccc} M_{aa} & M_{av} & M_{ap} & -C_{b}^{n} & 0_{3\times 3} & M_{ag} & 0_{3\times 6}\\ M_{va} & M_{vv} & M_{vp} & 0_{3\times 3} & C_{b}^{n} & 0_{3\times 9} & M_{vf}\\ 0_{3\times 3} & M_{pv} & M_{pp} & 0_{3\times 3} & 0_{3\times 3} & 0_{3\times 9} & 0_{3\times 6}\\ & & & 0_{21\times 30} & & & \end{array}\right]$$

The specific formulae of $F_{INS}$ are given in Appendix A.

According to the error model of INS, the positioning errors should grow at least quadratically over time. However, the matching algorithm usually executes once by using a dozen positioning points acquired within a very short period of time or in a small region, so that the INS indicated as trace can be regard as a linear transformation from the true trace during this short period. Then the relationship between indication trace $trace_{i}$ and real trace $trace_{t}$ can be described as Equation (6) in the matching region.

(6) $$trace_{i}=f_{S}(f_{R}(trace_{t}+\delta_{T}))$$

where $f_{S}$ is the scaling factor, $f_{R}$ is the rotating factor, and $\delta_{T}$ is the translation.

## 3. Geomagnetic Matching Algorithm

### 3.1. The Principle of ICCP Algorithm

**Figure 1:**
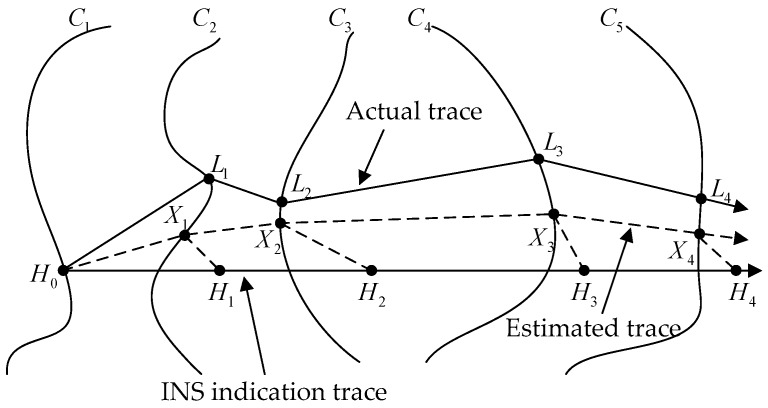
Illustration of inertial navigation system (INS) indication trace, Estimated trace, and Real trace.

The scenario for a UAV using the geomagnetic field to be located is such that the target starts from a position and travels along a 3D trace. Because that the altitude information of UAV can usually be given with high accuracy altimeter, Geomagnetic matching method only rectifies the indication trace of INS with 2D location points, as shown in Figure 1. Let us denote the indicated trace by $\left\{H_{i}\right\}$ (i=1,2,⋯,N) (*N* is the total number of points in matching region), which will be different from the actual trace $\left\{L_{i}\right\}$ (i=1,2,⋯,N) due to errors and drifts in instruments and random external effects referring to Equation (6). At the same time, the magnetic sensor provides the corresponding measured total geomagnetic intensity $\left\{g_{i}\right\}$ (i=1,2,⋯,N), and $\left\{C_{i}\right\}$ (i=1,2,⋯,N) is the corresponding set of geomagnetic field contour. If the actual trace $\left\{X_{i}\right\}$ (i=1,2,⋯,N) is known, the navigational errors can be corrected by a rigid transformation of the indicated trace into the actual trace. The rigid transformation *T* minimizes the distance between the sets $\left\{H_{i}\right\}$ and $\left\{X_{i}\right\}$ [17].

(7) $$Min\;E_{j}=\sum_{i=1}^{N}dis_{j}(X_{i}-X_{i-1},H_{i}-H_{i-1})+T\sum_{i=1}^{N}dis_{j}(X_{i},C_{i})$$

where $dis\left(X,H\right)={\left\|X-H\right\|}^{2}$. The extraction of the closest contour point is discussed in [16]. The minimization process of $E_{j}$ is iterated so that in the *j*-th iteration the set of measured points is $H^{j}=TH^{j-1}$ and the set of points $X^{j}$ is determined from the new points $H^{j}$. The iteration process is continued until *T* becomes negligible.

The rigid transformation *T* consists of the rotation matrix and the translation vector as shown in Equation (2), which can be solved with the quaternion method.

(8) $$H_{i+1}=\left(\begin{array}{l} t_{x}\\ t_{y} \end{array}\right)+\left(\begin{array}{ll} \cos\theta & -\sin\theta \\ \sin\theta & \cos\theta \end{array}\right)H_{i}$$

where θ refers to the rotation angle. $t_{x}$ and $t_{y}$ are translation.

### 3.2. VICCP Algorithm

In order to make full use of geomagnetic vector information, the rule of approaching of indicated trace toward closest contour points should be adjusted because there are three contours for the geomagnetic vector. We define an overall match error that must be minimized with respect to all $\left\{H_{i}\right\}$:

(9) $$\begin{array}{ll} E_{j} & =\sum_{i=1}^{N}d_{j}\left(X_{i}-X_{i-1},H_{i}-H_{i-1}\right)\\ & +T_{A}\sum_{k=1}^{3}\sum_{i=1}^{N}\lambda_{k}d_{j}\left(X_{i},\Omega \left(C_{i,k}\right)\right)/\sum_{i=1}^{N}\lambda_{k} \end{array}$$

where $\lambda_{k}$ is weighting coefficient, taking $\lambda_{k}=1$ without a priori knowledge. $\left\{C_{i,k}\right\}$ stands for the contour of each geomagnetic element. If the error model parameter of three-axis magnetometer is pre-determined, the geomagnetic vector can be corrected by inversing the measurement model with Equation (2).

(10) $$h_{E}=C_{M}^{-1}\left(h_{M}-d_{M}\right)$$

Then define corresponding contours extracted with the corrected geomagnetic vector as $\Omega \left(C_{i,k}\right)$.

Since we introduce more geomagnetic information in ICCP, a more complicated transformation can be adopted to improve the adaptability with the error characteristics of INS described with Equation (6), and without causing the divergence of the algorithm. The simplified affine transformation *T_A_* is used in this paper to solve the problem that traditional ICCP cannot reduce the scaling error of indication track in INS. Defining rotation matrix *R*, translation *t* and scale factor *S*, the matching process of the indication track sequence $H_{i}$ to the real track sequence $L_{i}$ of each iterative can be performed with Equation (11).

(11) $$L_{i}=SRH_{i}+t$$

The least squares solution of this transformation can be given by solving the Procrustes problem [18]. The cost function can be written as:

(12) $$\begin{array}{cc} f{\left(S,R,t\right)}_{i} & =\sum_{i=1}^{n}{\left\|L_{i}-\left(SRH_{i}+t\right)\right\|}^{2}\\ & =n{\left\|L_{0}-\left(SRH_{0}+t\right)\right\|}^{2}+\sum_{i=1}^{n}{\left\|L_{i}^{\prime }-\left(SRH_{i}^{\prime }+t\right)\right\|}^{2} \end{array}$$

where $L_{0}$ and $H_{0}$ denote the centroids of $L_{i}$ and $H_{i}$, $L_{i}^{\prime }$ and $H_{i}^{\prime }$ is the relative vectors relative to their centroids. Then normalize $L_{i}$ and $H_{i}$:

(13) $$L_{s,i}=L_{i}/\sqrt{\sum L_{0}^{2}},\;H_{s,i}=H_{i}/\sqrt{\sum H_{0}^{2}}$$

Define the matrix $A=L_{s}H_{s}^{T}$, where *L_s_* = [*L_s_*_,1_ *L_s_*_,2_…*L_s,n_*] and *H_s_* = [*H_s_*_,1_ *H_s_*_,2_…*H_s,n_*]. The least squares estimate of the rotation matrix *R* is Equation (14) when *A* is nonsingular.

(14) $$R=A{\left(A^{T}A\right)}^{-1/2}$$

In terms of the singular value decomposition of ***A*** as *UΛV^T^*, the optimal rotation matrix *R* can be expressed as:

(15) $$R=V\left[\begin{array}{ccc} 1 & 0 & 0\\ 0 & 1 & 0\\ 0 & 0 & \det\left(U\right)\det\left(V\right) \end{array}\right]U^{T}$$

The scale factor ***S*** is

(16) $$S=tr\left(\Lambda \right)\cdot \sqrt{\sum L_{0}^{2}}/\sqrt{\sum H_{0}^{2}}$$

Then minimize the first term contribution in Equation (12) by

(17) $$t=L_{0}-SRH_{0}$$

## 4. Simulation

**Figure 2:**
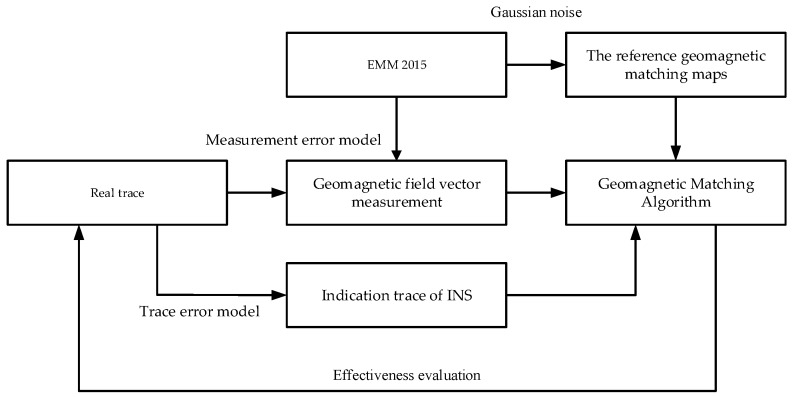
The simulation flow of geomagnetic matching navigation.

The simulation flow is as shown in Figure 2, and the effectiveness of the proposed matching method can be examined by this numerical simulation. The reference matching maps are generated with Enhanced Magnetic Model (EMM 2015) of order 720, and disturbed by a certain level of Gaussian noise. The simulated magnetic vector data are acquired based on the geomagnetic reference field and disturbed by inherent sensor errors and external interferences, with key parameters shown in Table 1. The INS simulation in this paper is carried out for a route under the condition shown in Table 2. The condition includes initial errors and measurement error. Note that this simulation only reveals the geomagnetic aiding process in the matching region, where the INS already has a large deviation before entering into the match regions.

### 4.1. The Comparison of ICCP and VICCP

**Figure 3:**
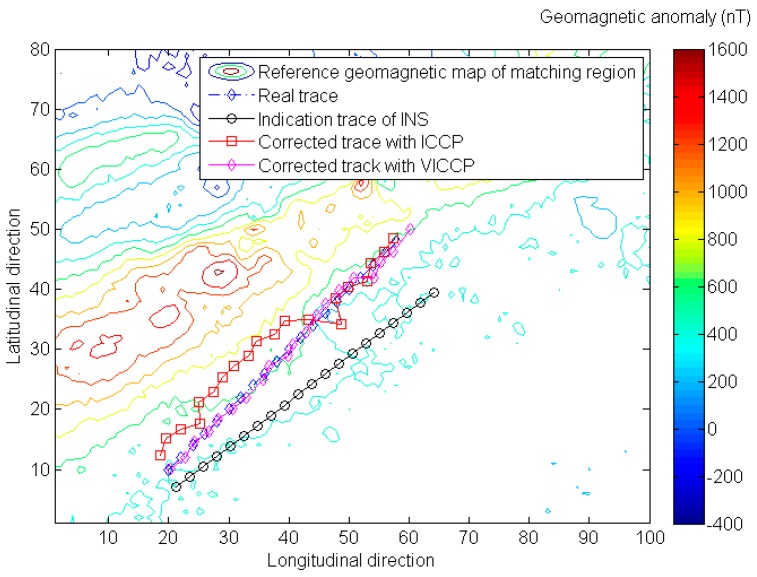
The comparison of geomagnetic matching results with iterative closest contour point (ICCP) and vector iterative closest contour point (VICCP). Note that the indication trace of INS does not contain scale error in this case.

Simulation results of introducing geomagnetic vector into VICCP algorithm shows that the vector matching method has better positioning precision and matching probability than the traditional ICCP when the indication trace of INS does not contain scale error, as shown in Figure 3 and Table 3.

**Figure 4:**
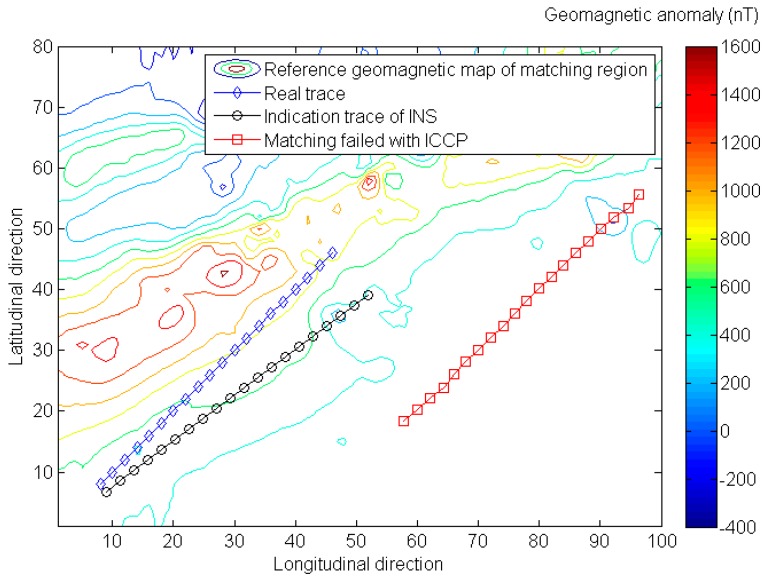
A considerable matching error will generate geomagnetic matching fails.

The matching probability means the percentage of average positioning errors less than the matching tolerance (200% of indication trace’s mean error, as shown in Table 1) in the Monte Carlo simulation. Actually, whenever the matching procedure fails, a considerable matching error will be generated. As shown in Figure 4, the mean positioning is around 5 km when the ICCP matching procedure fails. So the statistical quantities of positioning precision are given in the Tables below only when the matching procedure is successful.

### 4.2. The Validity of Affine Transformation Based VICCP

**Figure 5:**
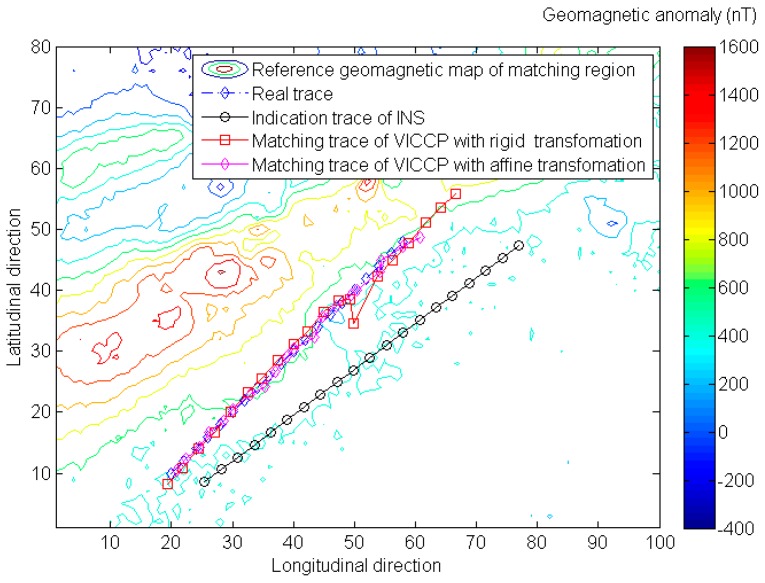
The comparison of geomagnetic matching results for VICCP with rigid transformation and affine transformation.

Simulation results in Figure 5 and Table 4 show that when scale error exists, the traditional ICCP algorithm becomes invalid, and the positioning accuracy and matching probability have been significantly reduced. In addition, the affine transformation based VICCP increases the adaptability with positioning error characteristics of INS, and achieves positioning accuracy of less than 200 m instead.

**Figure 6:**
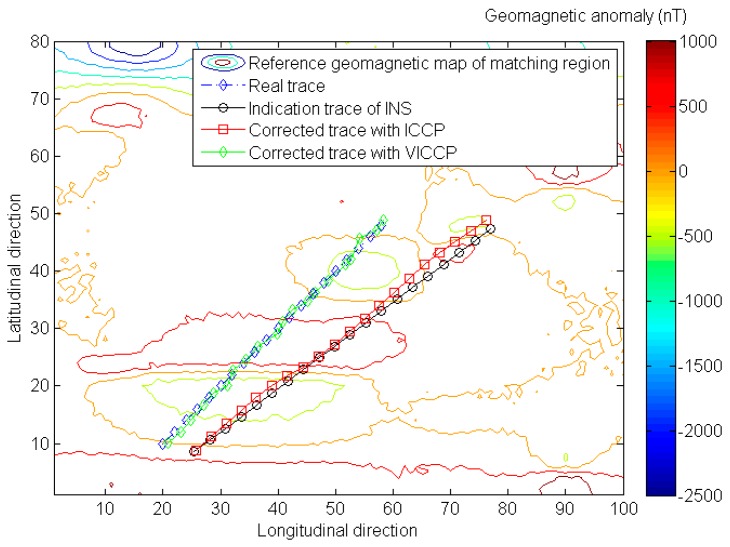
Simulation the effectiveness of proposed algorithm with the relative flat geomagnetic terrain.

In Figure 6, the effectiveness of proposed algorithm is evaluated with relative flat geomagnetic terrain, and the simulation results demonstrate the robustness of the proposed method. The geomagnetic entropy calculated with Equation (18) is much smaller than the matching region utilized in Figure 3.

(18) $$H_{X}=-\sum_{i=1}^{N}p_{j}\log p_{i},\;p_{i}=\left|h_{i}\right|/\sum_{i=1}^{N}h_{i}$$

where $h_{i}$ is the intensity of geomagnetic anomaly field.

### 4.3. The Comparison of VICCP and Bayesian Based VICCP

**Figure 7:**
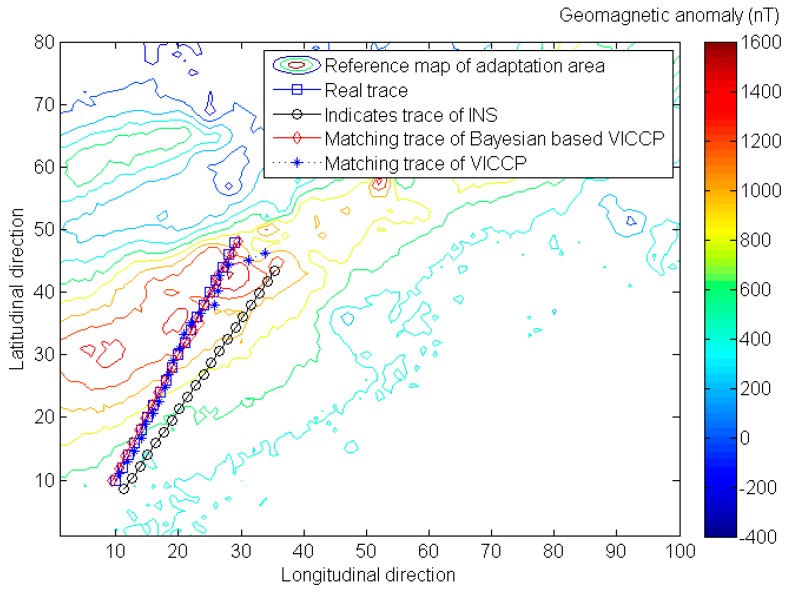
Evaluation the performance of Bayesian-based algorithm.

In order to further improve matching performance of vector matching method, a priori knowledge is required as a support. An improved algorithm based on the Bayesian statistical analysis can be derived with matching error expressed as:

(19) $$\begin{array}{cc} E_{j} & =\sum_{i=1}^{N}d_{j}\left(X_{i}-X_{i-1},H_{i}-H_{i-1}\right)\\ & +T_{A}\sum_{k=1}^{3}\sum_{i=1}^{N}p_{k}d_{j}\left(X_{i},\Omega \left(C_{i,k}\right)\right)/\sum_{i=1}^{N}p_{k} \end{array}$$

where $p_{k}$ is prior matching probability of each single element, which can obtain through experiment, and then $E_{j}$ in Equation (7) can be replaced with Equation (19). The Simulation result in Figure 7 and Table 5 show that the Bayesian-based algorithm will further improve the positioning accuracy and matching probability.

## 5. Conclusions

This paper proposed a geomagnetic matching method that makes full use of the geomagnetic vector information to improve accuracy and robustness. The achievable accuracy limits for the traditional matching algorithm were discussed, the affine transformation was adopted to ICCP for the first time to increase adaptability with positioning error characteristics of INS, and the best achievable positioning accuracy for geomagnetic matching error was less than 200 m in case of existing scale error in the indication trace. For better results, it is necessary to use prior knowledge such as matching probability of each single geomagnetic element, and achieves positioning error less than 100 m. In future work, we plan to extend this work to multi matching regions during a complete long range trajectory, a specific INS error model will be introduced rather than using a rough indication trace error model, and we will try to achieve 3D geomagnetic matching without altimeter aiding.

## Figures and Tables

**Table 1:** The parameters of simulation.

Parameter	Parameter Values
Matching points	20
Reference map noise	~(0, 25) nT
Matching region size	8 km × 10 km
Center location of matching region	(108.98° E, 34.91° N)
Average altitude	~20 km
Gird size of matching region	100 × 80
Measurement Gaussian noise	~10 nT/axis
Matching tolerance	200% of uncorrected trace’s mean error

**Table 2:** Simulation condition of INS.

Symbol	Quantity	Unit
Platform misalignment angles	30	″
Initial velocity error	0.1	m/s
Initial position error	10	m
Gyro constant bias	0.01	°/h
Gyro random walk	0.001	°/h
Accelerometor constant bias	100	μg
Accelerometor random walk	10	μg
Scale factor error	10	ppm
Askew installation error	10	″

**Table 3:** Comparison of ICCP and VICCP with 100 times Monte Carlo simulation.

Statistical Quantity	Indication Trace of INS	VICCP with Rigid Transformation	VICCP with Affine Transformation
Mean (m)	679.05	267.66	127.4
Var (m)	530.49	265.65	483.8
Matching probability (percentage)	-	93%	98%

**Table 4:** Comparison of VICCP with different transformation for 100 times Monte Carlo simulation.

Statistical Quantity	Indication Trace of INS	VICCP with Rigid Transformation	VICCP with Affine Transformation
Mean (m)	1225.14	600.20	153.16
Var (m)	1726.78	1142.51	50.07
Matching probability (percentage)	-	90%	98%

**Table 5:** Evaluation of the performance of the Bayesian based algorithm with 100 times Monte Carlo simulation.

Matching Method		Mean (m)	Var (m)	Matching Probability (percentage)
ICCP	X	235.37	240.28	88%
	Y	531.31	662.51	64%
	Z	185.42	201.27	92%
VICCP		124.72	51.05	97%
Bayesian-based VICCP		88.36	34.96	99%

## Appendix A

Assume the gyro sensed angular rate and accelerometer sensed specific force are $\omega_{ib}^{b}=[\begin{array}{ccc} \omega_{ibx}^{b} & \omega_{iby}^{b} & \omega_{ibz}^{b} \end{array}]$ and $f_{sf}^{b}=[\begin{array}{ccc} f_{sfx}^{b} & f_{sfy}^{b} & f_{sfz}^{b} \end{array}]$, then the components in Equation (5) are given as below:

(A1) $$M_{aa}=(-\omega_{in}^{n}\times )$$

(A2) $$M_{av}=\left[\begin{array}{ccc} 0 & -1/R_{Mh} & 0\\ 1/R_{Nh} & 0 & 0\\ \tan L/R_{Nh} & 0 & 0 \end{array}\right]$$

(A3) $$M_{ap}=\left[\begin{array}{ccc} 0 & 0 & v_{N}^{n}/R_{Mh}^{2}\\ -\omega_{ie}\sin L & 0 & -v_{E}^{n}/R_{Nh}^{2}\\ v_{E}^{n}\sec^{2}L/R_{Nh}+\omega_{ie}\cos L & 0 & -v_{E}^{n}\tan L/R_{Nh}^{2} \end{array}\right]$$

(A4) $$M_{ag}=-\left[\begin{array}{ccc} \omega_{ibx}^{b}C_{b}^{n} & \omega_{iby}^{b}C_{b}^{n} & \omega_{ibz}^{b}C_{b}^{n} \end{array}\right]$$

(A5) $$M_{va}=(f_{sf}^{n}\times )$$

(A6) $$M_{vv}=(v^{n}\times )M_{av}-((2\omega_{ie}^{n}+\omega_{en}^{n})\times )$$

(A7) $$M_{vp}=(v^{n}\times )\left[\begin{array}{ccc} 0 & 0 & v_{N}^{n}/R_{Mh}^{2}\\ -2\omega_{ie}\sin L & 0 & -v_{E}^{n}/R_{Nh}^{2}\\ v_{E}^{n}\sec^{2}L/R_{Nh}+2\omega_{ie}\cos L & 0 & -v_{E}^{n}\tan L/R_{Nh}^{2} \end{array}\right]+\left[\begin{array}{ccc} 0 & 0 & 0\\ 0 & 0 & 0\\ -g_{0}\beta_{1}\sin L\cos L & 0 & \beta_{3} \end{array}\right]$$

(A8) $$M_{vf}=\left[\begin{array}{cccc} f_{sfx}^{b}C_{b}^{n} & f_{sfy}^{b}C_{b}^{n}(:,2) & f_{sfy}^{b}C_{b}^{n}(:,3) & f_{sfz}^{b}C_{b}^{n}(:,3) \end{array}\right],\;C_{b}^{n}(:,i)\;=\;i\text{th column of }C_{b}^{n}$$

(A9) $$M_{pv}=\left[\begin{array}{ccc} 0 & 1/R_{Mh} & 0\\ \sec L/R_{Nh} & 0 & 0\\ 0 & 0 & 1 \end{array}\right]$$

(A10) $$M_{pp}=\left[\begin{array}{ccc} 0 & 0 & -v_{N}^{n}/R_{Mh}^{2}\\ v_{E}^{n}\sec L\tan L/R_{Nh} & 0 & -v_{E}^{n}\sec L/R_{Nh}^{2}\\ 0 & 0 & 0 \end{array}\right]$$

The “×” in $\omega_{in}^{n}\times$, $f_{sf}^{n}\times$, $v^{n}\times$, etc. is the skew-symmetric operator of the vector, and where $f_{sf}^{n}=C_{b}^{n}f_{sf}^{b}$, $\omega_{nb}^{b}=\omega_{ib}^{b}-{\left(C_{b}^{n}\right)}^{T}\omega_{in}^{n}$, and $\omega_{in}^{n}=\omega_{ie}^{n}+\omega_{en}^{n}$, with

(A11) $$\{\begin{array}{c} \omega_{ie}^{n}={\left[\begin{array}{ccc} 0 & \omega_{ie}\cos L & \omega_{ie}\sin L \end{array}\right]}^{T}\\ \omega_{en}^{n}={\left[\begin{array}{ccc} -\frac{v_{N}^{n}}{R_{Mh}} & \frac{v_{E}^{n}}{R_{Nh}} & \frac{v_{E}^{n}}{R_{Nh}}\tan L \end{array}\right]}^{T} \end{array}$$

(A12) $$\{\begin{array}{c} R_{Mh}=R_{M}+h,\;R_{Nh}=R_{N}+h\\ R_{M}=\frac{R_{N}(1-e^{2})}{\left(1-e^{2}\sin^{2}L\right)},\;R_{N}=\frac{R_{e}}{{\left(1-e^{2}\sin^{2}L\right)}^{1/2}}\\ e=\sqrt{2f-f^{2}} \end{array}$$

(A13) $$\{\begin{array}{c} g^{n}={\left[\begin{array}{ccc} 0 & 0 & -g \end{array}\right]}^{T}\\ g=g_{0}(1+\beta_{1}\sin^{2}L+\beta_{2}\sin^{4}L)-\beta_{3}h, \end{array}\mathrm{with}\{\begin{array}{c} \beta_{1}=5.27094\times 10^{-3}\\ \beta_{2}=2.32718\times 10^{-5}\\ \beta_{3}=2g_{0}/R_{e}=3.086\times 10^{-6}(1/s^{2}) \end{array}$$

$C_{b}^{n}$ is transformation direct cosine matrix from body frame to navigation frame; $v^{n}={\left[\begin{array}{ccc} v_{E}^{n} & v_{N}^{n} & v_{U}^{n} \end{array}\right]}^{T}$ is velocity along east, north and up-vertical direction; $p={\left[\begin{array}{ccc} L & \lambda & h \end{array}\right]}^{T}$, where L,λ,h is latitude, longitude and altitude; $R_{e}=6371.2\text{ km}$ is the Earth’s semi-major axis; f=1/298.257 is the Earth’s flattening; $\omega_{ie}=7.2921151467\times 10^{5}$ rad/s is the Earth’s angular rate; $g_{0}=9.7803267714\;{m/s}^{2}$ is gravity magnitude at the equatorial sea-surface.

## References

[1] Tyren C. (1982). Magnetic anomalies as a reference for ground-speed and map matching navigation. J. Navig..

[2] Tyren C. Magnetic terrain navigation. Proceedings of the 5th International Symposium on Unmanned Untethered Submersible Technology.

[3] Peng W., Wu Y., Hu X., Ruan Q., Yuan H. Geomagnetic aided navigation suitability evaluation based on principal component analysis. Proceedings of the 2012 International Conference on Industrial Control and Electronics Engineering (ICICEE).

[4] Yang G., Li S., Jiang Z. (2007). Data fusing algorithm in geomagnetic aided INS. J. Chin. Inertial Technol..

[5] Mu H., Wu M., Hu X., Ma H. Geomagnetic surface navigation using adaptive EKF. Proceedings of the 2nd IEEE Conference on Industrial Electronics and Applications.

[6] Ejaz M., Iqbal J., Ahsan N., Nawaz A. Robust geomagnetic aided inertial navigation of underwater vehicles using the ICP algorithm. Proceedings of the Asia-Pacific Conference on Computational Intelligence and Industrial Applications.

[7] Zhao J., Wang S., Wang A. Study on underwater navigation system based on geomagnetic match technique. Proceedings of the 9th International Conference on Electronic Measurement & Instruments (ICEMI ‘09).

[8] Ren Z., Chen L., Zhang H., Wu M. Research on geomagnetic-matching localization algorithm for unmanned underwater vehicles. Proceedings of the International Conference on Information and Automation (ICIA 2008).

[9] Wei L., Zhi W., Wu M., Hu X. Geomagnetic matching technology based on Iterative Contour Matching algorithm. Proceedings of the 10th International Conference on Electronic Measurement & Instruments (ICEMI).

[10] Zhou J., Liu Y., Ge Z. (2011). Geomagnetic matching algorithm based on the probabilistic neural network. Proc. Inst. Mech. Eng. Part G: J. Aerosp. Eng..

[11] Kauffman K., Raquet J. Navigation via H-field signature map correlation and INS integration. Proceedings of the 2014 IEEE Radar Conference.

[12] Včelák J., Ripka P., Zikmund A. (2015). Precise magnetic sensors for navigation and prospection. J. Supercond. Novel Magn..

[13] Ripka P. Magnetic sensors for navigation and security applications. Proceedings of the 2006 IEEE Instrumentation and Measurement Technology Conference Proceedings.

[14] Renaudin V., Afzal M.H., Lachapelle G. (2010). Complete triaxis mag-netometer calibration in the magnetic domain. J. Sens..

[15] Unsal D., Demirbas K. Estimation of deterministic and stochastic IMU error parameters. Proceedings of the 2012 IEEE/ION Position Location and Navigation Symposium (PLANS).

[16] Titterton D., Weston J. (1997). Strapdown Inertial Navigation Technology.

[17] Kamgar-Parsi B., Kamgar-Parsi B. (1999). Vehicle localization of gravity maps. Proc. SPIE.

[18] Dorst L. (2005). First order error propagation of the procrustes method for 3D attitude estimation. IEEE Trans. Pattern Anal. Mach. Intell..

